# Removal of a Portion of the Left Lung

**Published:** 1855-02

**Authors:** T. B. Hale


Removal of a Portion of the left Lung. By T. B. Hale, M. D.—C. D——, an Irishman, aged 25 years, rather small in stature, but stoutly built, with a well developed chest, being engaged in a fight while intoxicated, received a stab in the left side, parallel with the ribs. The wound was about one inch and a quarter long, and appeared to have been made with a sharp, clean-cutting instrument. About fourteen hours after the injury he was visited by Dr. Hale, who found, upon examination, a portion of the left lung protruding from the thorax. He was sitting up in bed, having the protruded portion supported by a broad bandage. He complained of no pain, and had suffered but little from loss of blood. There was no cough or difficulty of breathing, but on taking a full inspiration the protruded lung became filled with air, and drops of venous blood oozed from its substance. The protrusion was so tightly strangulated at the wound in the thorax that after an hour and a half spent in unsuccessful efforts to restore it, Dr. Hale made a cautious attempt to enlarge the wound in the interosseous space. Fearing, however, the effect of a large opening into the cavity of the pleura, he was induced to desist, and consider the propriety of excision. As the protrusion looked extremely unhealthy, from the length of time since the accident and the efforts made to reduce it, making gangrene not an improbable result, excision seemed to be the only resource. Dr. Hale contemplated applying a ligature at the base of the protruded lung, but on making two experimental incisions into its substance, and no blood flowing, this was not judged necessary, but the mass was at once excised, and the remaining portion pushed back through the wound in the interosseous space, the orifice of which was then closed with two stitches and strips of adhesive plaster. The patient was then directed to lie quietly on his back, and a mixture of two parts syr. prun. virgin., and one part syr. opii prescribed; a tablespoonful to be given every two hours for the purpose of allaying irritation in the bronchial tubes. On the second day Dr. Hale found him in a favorable condition, and on the sixth day he walked five miles to visit his physician, suffering in no manner from the loss of the portion of lung. For the last three months he has labored constantly in the coal mines, without inconvenience.

The speedy recovery of the patient appears to have been due to adhesive inflammation between the adjacent walls of the pleura, through the wound in which the protruded lung was strangulated. In all probability the pulmonary and costal pleura and the substance of the lung are all connected in the same cicatrix.

